# Ginsenoside Rg3 micelles mitigate doxorubicin-induced cardiotoxicity and enhance its anticancer efficacy

**DOI:** 10.1080/10717544.2017.1391893

**Published:** 2017-10-24

**Authors:** Lan Li, Jingyu Ni, Min Li, Jingrui Chen, Lifeng Han, Yan Zhu, Deling Kong, Jingyuan Mao, Yi Wang, Boli Zhang, Meifeng Zhu, Xiumei Gao, Guanwei Fan

**Affiliations:** a First Teaching Hospital of Tianjin University of Traditional Chinese Medicine, Tianjin, PR China;; b State Key Laboratory of Modern Chinese Medicine, Tianjin University of Traditional Chinese Medicine, Tianjin, PR China;; c State Key Laboratory of Medicinal Chemical Biology, Key Laboratory of Bioactive Materials of Ministry of Education, College of Life Science, Nankai University, Tianjin, PR China;; d College of Pharmaceutical Sciences, Zhejiang University, Hangzhou, PR China

**Keywords:** Doxorubicin, cardiotoxicity, Ginsenoside Rg3, anticancer efficacy, micellar drug delivery

## Abstract

Doxorubicin (DOX) is one of the most effective chemotherapy agents used in the treatment of hematological and solid tumors, however, it causes dose-related cardiotoxicity that may lead to heart failure in patients. One of the major reasons was increased reactive oxygen species (ROS) production. Ginsenoside Rg3 (Rg3), was powerful free radical scavengers and possessed cardioprotective effects. Nevertheless, Rg3 has low aqueous solubility and oral bioavailability, limiting its effects. Herein, we encapsulated Rg3 through spontaneous self-assembly of Pluronic F127 to improve its solubility and oral bioavailability. Moreover, co-administering Rg3 in Pluronic F127 micelles with doxorubicin can mitigate the cardiotoxicity, with ameliorating mitochondrial and metabolic function, improving calcium handling, and decreasing ROS production. In addition, it can improve the anticancer efficacy of doxorubicin. Therefore, our study provides a rational strategy for further developing a potentially viable adjunct-supportive treatment for reducing toxicity and increasing efficiency on chemotherapy.

## Introduction

Doxorubicin (DOX) is one of the most effective clinical anticancer drugs, which has been widely used for the treatment of various hematological and solid tumors (Lipshultz et al., 2015). However, in clinical applications, it exhibits dose-related cardiotoxic side effects, which may compromise the efficacy of chemotherapy and have a serious impact on the patient’s survival time, and quality of life (Giordano et al., 2012). The mechanism of DOX-induced cardiotoxicity (DIC) is complicated and may involve several interconnected events, including reactive oxygen species (ROS) generation, mitochondrial dysfunction, cell membrane injury and apoptosis, and calcium overloading (Scott et al., 2011; Octavia et al., 2012; Zhang et al., 2012). Researchers have been committed to screening and identifying drugs to mitigate cardiotoxicity.

Numerous natural products reportedly possess particular health-promoting and therapeutic functions *in vitro* and *in vivo* (Tang et al., 2016; Hao et al., 2017). Ginsenoside Rg3 (Rg3), a main active constituent of *Panax ginseng*, exhibits a range of pharmacological effects including antioxidant, immunomodulatory, anti-inflammatory, and anti-aging activities in several disease (Cheng et al., 2013a; Cheng et al., 2014; Smith et al., 2014; Cheng et al., 2016a). In addition, several studies have also shown that Rg3 exhibits cardioprotective and anticancer activities (Wang et al., 2015b; Zhou et al., 2016). These findings suggest that DOX administered with Rg3 will not only antagonize DIC but also improve the anticancer effect. However, Rg3 has low solubility and oral bioavailability (Yang et al., 2012), which limits its effects.

Pluronic F127 (PF127) has been widely studied in biomedical and pharmaceutical sciences (Hou et al., 2016; Luo et al., 2016). It involves triblock copolymers comprising a polypropylene oxide (PPO) chain conjugated with two polyethylene oxide (PEO) chains on both sides and can spontaneously self-assemble into polymeric micelles. While the PPO segment constitutes a hydrophobic core as a microenvironment for lipophilic drug encapsulation, the PEO segment inhibits protein adsorption, which can increase the solubility of an insoluble drug (Batrakova & Kabanov, 2008; Xu et al., 2012). The PF127 nano-carrier system is easy to prepare and avoids the use of organic solvents, enabling site-specific delivery. Several studies have demonstrated that Pluronic could be used as a multidrug resistance (MDR) modulator (Sarisozen et al., 2012; Chen et al., 2013), and it could help the drug to cross the blood–brain barrier and intestinal barriers and prevent the incorporated proteins from being eliminated by the reticuloendothelial system (RES) (Sonali Agrawal et al., 2016; Meng et al., 2017). Pluronic micelles could also extend circulation times for the encapsulated molecules *in vivo* (Chen et al., 2014). Presently, a Pluronic-based micellar delivery system for DOX is in Phase III clinical trial (Oerlemans et al., 2010). Therefore, we propose encapsulating Rg3 through the spontaneous self-assembly of Pluronic F127 to improve its aqueous solubility and oral bioavailability.

The clinical application of combined medication has been shown to reduce the toxic side effects and enhance drug therapeutic efficacy (Sleijfer et al., 2005; Cote et al., 2015). Here, we hypothesize that a combination of Rg3-loaded Pluronic F127 micelles and DOX will mitigate cardiotoxicity while improving the anticancer efficacy of the latter. To verify this hypothesis, we evaluated the cardioprotective and anticancer effects of Rg3-loaded Pluronic F127 micells co-administered with DOX using *in vivo* and *in vitro* models, and further investigated the mechanism of this combination therapy to demonstrate the performance of the medication strategy.

## Methods

### Animals

Sprague–Dawley (SD) male rats (200 ± 20 g), male and female C57/BL mice (18 ± 2 g), and male and female Balb/C mice (20 ± 2 g) were all provided by Beijing Vital River Laboratory Animal Technology Co., Ltd. (Beijing, China). The animals were housed in a specific-pathogen-free (SPF) animal facility of Tianjin University of Traditional Chinese Medicine, under controlled temperature 22 ± 2 °C and humidity 40 ± 5% with a 12-h light/dark cycle, and received standard diet and water. Three different sets of animals were used: (1) SD male rats were used for *in vivo* pharmacokinetic study; (2) C57/BL mice were used for *in vivo* assessment of the cardioprotective effect of Rg3-loaded Pluronic F127 micelles at a toxic DOX dosing. (3) Balb/C mice were used for *in vivo* assessment of tumor weight. The animals were fasted for 12 h before the experiment, but allowed free access to water. The experimental procedures conformed to directive 2010/63/EU of the European Parliament and all animals were handled in accordance with the guidelines of Tianjin University of Traditional Chinese Medicine Animal Research Committee (TCM-LAEC2014005).

### Preparation and characterization of Rg3-loaded Pluronic F127 micelles (P-Rg3)

Stock solutions containing 200 mg of Pluronic-F127 (Sigma-Aldrich, St. Louis, MO) and 10 mg of Rg3 (Shanghai Winherb Medical Technology Co., Ltd., Shanghai, China) were combined and added to 15 mL of ethanol. A polymer film was formed in 60 °C water bath over 8 min. After cooled to room temperature, it was rehydrated in 5 mL deionized water. The solution was filtered using a 0.22-μm nylon filter. The size of P-Rg3 was verified by dynamic light scattering (DLS) by diluting fresh P-Rg3 with deionized water to a final concentration of 1 mg/mL. The mean size and polydispersity index (PDI) for three replicates are obtainable. Freshly prepared P-Rg3 was diluted in acetonitrile to a final polymer concentration of 0.1 mg/mL and was assessed for drug-loading and encapsulation ratios. Rg3 in micelles was quantified using an Agilent 2460 Series HPLC equipped. Agilent Eclipse plus C18 RRHD column (2.1 × 50 mm, 1.8 μm) was maintained at 40 °C. The mobile phase consisted of water:acetonitrile (1:1). The flow rate was maintained at 1 mL/min and an injection of 20 μL was used with an 11 min run time. Rg3 peak area was assessed at 203 nm using a DAD detector at a retention time of 9.23 min. Data are presented as mean loading ± SD for three replicates. Encapsulation ratios and also drug-loading coefficients were calculated using the following equations:

$$\text{ER\%}=\frac{\text{Weight }\text{of }\text{the }\text{drug }\text{in }\text{micelles}}{\text{Weight }\text{of }\text{the }\text{feeding }\text{drug}}\times 100\%$$

$$\text{DL\%}=\frac{\text{Weight }\text{of }\text{the }\text{drug }\text{in }\text{micelles}}{\text{Weight }\text{of }\text{the }\text{feeding }\text{polymer }\text{and }\text{drug}}\times 100\%$$

### *In vitro* drug release from P-Rg3

Freshly prepared P-Rg3 samples with a volume of 10 mL were placed in dialysis cassettes with a molecular weight cutoff of 3 KDa. The cassettes were placed in 400 mL of 0.2 M phosphate buffer (pH7.4) and maintained at 37 °C for 72 h. Then, 1-mL samples were withdrawn at 0, 0.084, 0.167, 0.25, 0.5, 1, 2, 3, 6, 8, 10, 12, 24, 36, 48, and 72 h. Simultaneous equal volume buffer were replaced. The collected samples were diluted to 1:1 in methanol and quantified using HPLC.

### Pharmacokinetic study

Twelve Sprague–Dawley male rats (200 ± 20 g) were provided by Beijing Vital River Laboratory Animal Technology Co., Ltd. (Beijing, China). The rats were assigned randomly into two groups: Ig administration of P-Rg3 or Ginsenoside Rg3 solution, with six rats for each group. Rats were made to fast for 12 h before dosing and were allowed free access to water. A single oral dose of 50 mg/kg P-Rg3 and Ginsenoside Rg3 solution prepared with physiological saline was administered to each rat. Rat blood samples (250 μL) were collected in heparinized 1.5-mL polythene tubes via the suborbital vein at 0, 0.033, 0.083, 0.167, 0.25, 0.333, 0.5, 1, 2, 4, 6, 8, 10, 24, 34, and 48 h after oral administration. The blood samples were separated by centrifugation at 7000 *g* for 10 min and stored at −20 °C until analysis. The LC/MS analysis was performed on an Ultimate 3000 UHPLC coupled with a Q-Exactive mass spectrometer by HESI source (Thermo Fisher Scientific, Co., Waltham, MA). Chromatographic separation was achieved on an Agilent Eclipse plus C18 RRHD column (2.1 × 50 mm, 1.8 μm). The mobile phase was water (containing 0.05% acetic acid, *v*/*v*) (A) and ACN (D). Gradient elution was set as follows: 20–100% D from 0 to 4.0 min, 100–20% D from 4.0 to 4.5 min, and 20% D from 4.5 to 5.0 min. The flow rate was set at 0.4 mL/min and the injection volume was 2 μL. The column temperature was maintained at 40 °C. Source parameters were optimized as follows: SIM scanning in negative mode was used; the source spray voltage was set at 2.8 kV; capillary and Aux gas heater temperatures were 320 and 350 °C; and S-lens RF level, sheath gas (N_2_), and auxiliary gas (N_2_) were 50, 35, and 10 arbitrary units, respectively. Quasi-molecule ions of [M − H] were selected at *m/z* 783.49002 for Ginsenoside Rg3 and *m/z* 799.48493 as per the internal standard. The pharmacokinetic data were analyzed using Drug and Statistics version 1.0 (DAS; Medical College of Wannan, Anhui Sheng, China).

### *In vivo* assessment of cardioprotective effect of P-Rg3 in toxic DOX dosing

A mixed population of male and female C57/BL mice was divided into five groups (10 mice for the control group and 30 mice for the other groups). DOX is known to cause acute cardiotoxicity when administered at a dose of 15 mg/kg or greater (Walker et al., 2011). In this study, DOX was administered via a single intraperitoneal dose of 20 mg/kg in the DOX, DOX + PF127, DOX + Rg3, and DOX + P-Rg3 groups. The control group received normal saline. After the injection of DOX, Rg3 and P-Rg3 were prepared by dissolving the drug in normal saline, and were administered with 10 mg/kg Rg3 per day over 14 d. Mice in the control and DOX groups were treated with saline instead, and with PF127 200 mg/mL stock solution in the DOX + PF127 group. Cardiac left ventricular function and endocardial peak velocity were evaluated by Doppler imaging (Vevo 2100 ultrasound system; Visualsonics, Toronto, Canada). Plasma was separated by centrifugation at 3000 rpm for 15 min immediately after collection for biochemical estimations. The plasma was used to detect ANP and BNP using an enzyme-linked immunosorbent assay kit (ELISA, Cloud-Clone Corp, Katy, TX) following the manufacturer’s instructions. The levels of creatine kinase (CK), creatine kinase-MB (CK-MB), and lactate dehydrogenase (LDH) in serum were assessed by an automatic biochemical detector (Thermo Fisher Scientific), in accordance with the manufacturer’s instructions. Heart tissues were fixed in 4% paraformaldehyde for more than 48 h. Tissues were embedded in paraffin, cut into 4-µM sections, and subjected to hematoxylin eosin staining (H&E). Pictures were taken from three random areas from three sections per mouse using a digital camera connected to an optical microscope.

### Cell culture and viability studies

4T1β cells, MDA-MB-231 cells, and H9C2 cells were planted at 5000, 5000, and 10,000 cells/well in a 96-well flat-bottomed plate, respectively, and cultured for 24 h at 37 °C and 5% CO_2_. Cells were treated with Rg3, PF-127, and DOX in DMSO. The final concentration of DMSO in the wells was 1‰. Cells were also treated with P-Rg3 diluted in normal saline. The concentration of DOX was 3 μM and that of the natural products was 10 μM in H9C2 cells. The concentration of DOX was 1 μM and that of the natural products was 10 μM in 4T1β and MDA-MB-231 cells. Upon treatment, the plates were incubated for 48 h. Cell viability was assessed using MTT assay. For the Caspase-Glo 3/7^®^ assay, cells were seeded in accordance with the instructions of the cell viability assay in opaque 96-well plates. Luminescence was measured in accordance with the manufacturer’s instructions.

### Transmission electron microscopy (TEM)

For TEM, heart tissues and H9C2 cells were processed for ultrastructural analysis using electron microscopy (Kirshenbaum & Singal, 1992). Left ventricles were cut into three 3 × 1 × 1 mm^3^ random areas of the cubes. Heart cubes and H9C2 cells were fixed in 2% glutaraldehyde. Then, they were osmicated in 2% OsO_4_, after which ultrathin sections were stained with uranyl acetate and lead citrate, and then subjected to ultrastructural examination. Pictures were taken from three random areas from three sections per mouse or three independent cell experiments.

### Terminal dUTP nick end-labeling (TUNEL)

TUNEL assay was performed in accordance with the manufacturer’s protocol (Roche Diagnostics, Basel, Switzerland). Pictures were taken from three random areas from three sections per mouse or three independent cell experiments using a digital camera connected to an optical microscope or equipped with a fluorescence microscope.

### Isolation of mitochondria

The isolation of intact mitochondria from heart tissue and H9C2 was performed as previously described (Chen et al., 2015). Tissues or cells were used for mitochondrial isolation using mitochondrial extraction kit (Solarbio, China), and the remaining samples were quickly frozen in liquid nitrogen. Protein content of the mitochondria was quantified with Coomassie Brilliant Blue (Jianchen, Shanghai, China), in accordance with the manufacturer’s instructions.

### Measuring changes in mitochondrial function

Isolated mitochondria were planted in a 96-well flat-bottomed plate. The intramitochondrial ROS level was measured using 2′, 7′-dichlorofluorescein diacetate (DCFH-DA) fluorescent probe detection kit (Thermo Fisher Scientific). Mitochondrial inner membrane potential (ΔΨm) was monitored by applying the fluorescent dye JC-1 (Beyotime, China). Mitochondrial calcium loading in cardiac myocytes in the absence and presence of DOX was assessed by Fluo-4 AM (Thermo Fisher Scientific). Quantification of myocardial mitochondrial ATP content was accomplished with ATP Bioluminescent Assay Kit (Beyotime, China).

### Mitochondrial respiration

Mitochondrial oxygen consumption rate (OCR) was assessed with a Seahorse XF24 analyzer. *In vitro* H9C2 cells were cultured in 24-well plates, followed by the sequential addition of 1 μM oligomycin, 1 μM FCCP, and 1 μM and rotenone combined with 1 μM antimycin. For *in vivo* mitochondrial respiration, mitochondria were isolated from mouse hearts as described above and analyzed for respiration with the Seahorse analyzer (Rogers et al., 2011).

### Real-time reverse transcriptase polymerase chain reaction (RT-PCR) quantification and mitochondrial DNA quantification

RT-PCR analysis was performed to determine the expression of ANP, BNP, ANF, α-SKA, β-MHC, Bax, and Bcl-2 on heart tissues or cell lysate extract. Quantification of mitochondrial DNA was performed by RT-PCR, and the RT-PCR values were corrected to GAPDH expression levels and normalized with respect to baseline controls.

### Western blot analysis

Western blot analysis was performed to determine the expression of oxidative phosphorylation (OXPHOS) complexes, cytochrome-c (Cyc-C), ATP synthase delta 5 (ATP5D), and mitochondrial uncoupling protein 3 (UCP3), Caspase3, and Caspase9 on heart tissues or cell lysate extract. The relative values were corrected to GAPDH expression levels and normalized with respect to baseline controls.

### *In vivo* assessment of tumor weight

A total of 1 × 10^6^ 4T1β cells in 50 μL (by volume) of PBS were introduced by subcutaneous injection into the right flank. When the tumor reached approximately 240–280 mm^3^ in size, mice were divided into five groups (15 mice for each group). DOX was administered at 3 mg/kg via intraperitoneal injection every 3 d 4 times in the DOX, DOX + PF127, DOX + Rg3, and DOX + P-Rg3 groups. Meanwhile, Rg3 and P-Rg3 were prepared by dissolving the drug in normal saline, and were administered with 10 mg/kg Rg3 per day over 14 d in the DOX + Rg3 and DOX + P-Rg3 groups. Mice in the control groups were treated with saline instead, and with PF127 200 mg/mL stock solution in the DOX + PF127 group. Then, tumor weight was measured after the 14-d dosing period.

### Statistical analysis

All quantitative results were obtained from at least three samples for analysis. Data are expressed as the mean ± SD. Multiple comparisons between multiple groups were performed using one-way ANOVA. Significance level was accepted at a *p* value below .05 and .01.

## Results

### Preparation and characterization of Rg3-loaded Pluronic F127 micelles (P-Rg3)

**Figure 1.**
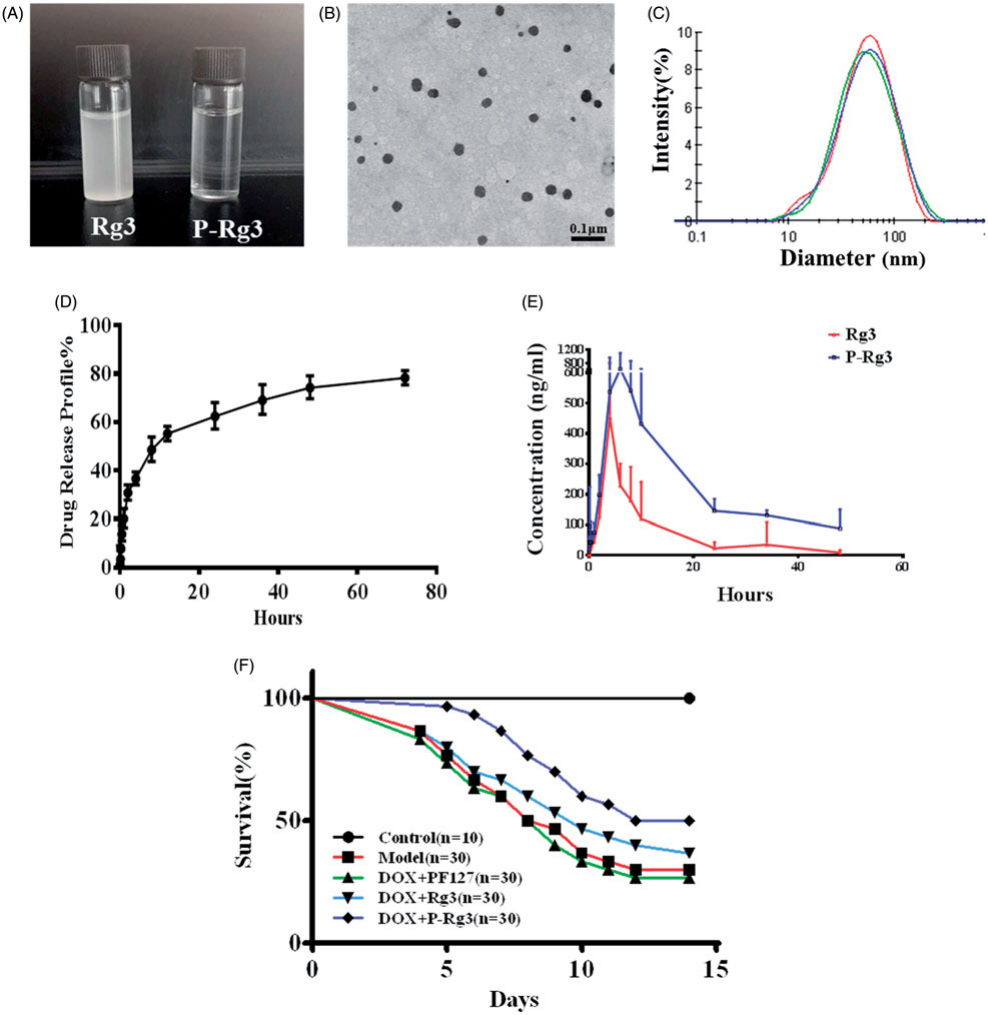
Characterization of Rg3-loaded Pluronic F127 micelles (P-Rg3) and the plasma concentration–time as well as survival profile curve. (A) Rg3 had poor water solubility and its solution was turbid, while P-Rg3 had high water solubility and its solution was clear and transparent. (B) Transmission electron microscopy (TEM) image showed the morphology of P-Rg3. (C) Size distribution of P-Rg3 was detected by dynamic light scattering (DLS). (D) Release profiles of P-Rg3 in PBS (PH 7.4) medium (*n* = 3). (E) The mean plasma concentration–time curve of Rg3 and P-Rg3 after oral administration for more than 48 h (*n* = 6). (F) Survival curves of each group (*n* = 10 for the control group and *n* = 30 for the other groups).

To detect the effect of PF127 on Rg3 encapsulation, drug encapsulation ratio (ER%) and loading coefficient (DL%) for P-Rg3 were first evaluated in this study. ER% and DL% of Rg3-loaded micelles were 96.47 ± 1.92% and 4.59 ± 0.05%, respectively, which indicated that Pluronic F127 micelles have acceptable ER% and DL% for Rg3. Accordingly, solubility of P-Rg3 was increased by 50–200 times compared with its intrinsic solubility. The Rg3 was suspended in water, while the P-Rg3 was almost completely soluble in water and its solution was transparent (Figure 1(A)). The enhancement of solubility provided by the formulation allowed us to assess these natural products at clinically relevant concentrations. We also characterized the micromorphology of P-Rg3. The TEM image showed that P-Rg3 were nanoparticles with uniform size (Figure 1(B)). Particle size directly affects the circulation time and biodistribution of the drug. Therefore, we tested the size of the P-Rg3 in water. DLS detection showed that the micelles had a uniform particle size distribution, and the size of micelles was 49.44 ± 0.15 nm, with an acceptable polydispersity index (PDI =0.339 ± 0.001) (Figure 1(C)). The process of drug release was initially rapid and then slowed, with the release ratio reaching approximately 80% at 72 h (Figure 1(D)). We also investigated the bioavailability of P-Rg3. After oral administration at a dosage of 50 mg/kg, the area under the time curve (AUC) of P-Rg3 was approximately 3.2 times greater than that of Rg3 in rat plasma, which indicated that the encapsulation of Ginsenoside Rg3 could significantly enhance its bioavailability (Figure 1(E)). P-Rg3 administration effectively reduced mortality caused by DOX; at 14 d, the mortality rate was 70.0% in the DOX group, 60% in the Rg3 group, and 45% in the P-Rg3 group, while no mortality was observed in the control group (Figure 1(F)).

### P-Rg3 improved cardiac function in response to DIC

**Figure 2.**
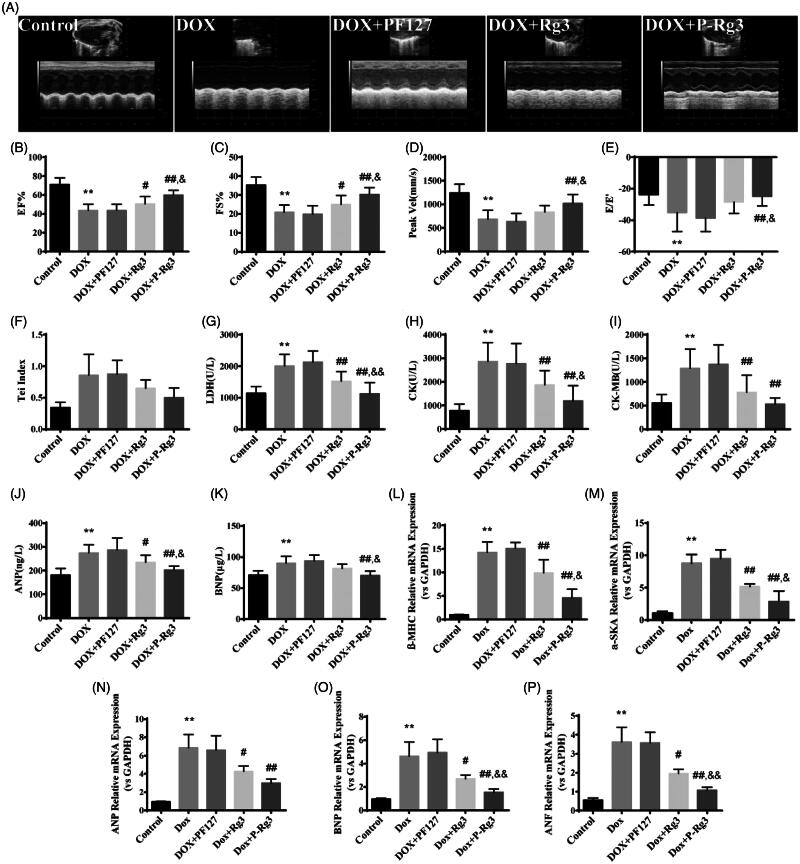
P-Rg3 ameliorated cardiac dysfunction induced by DOX. (A) Representative M-mode echocardiograms of mice in each group. (B–F) Left ventricular ejection fraction (EF %), fractional shortening (FS %), aortic valve peak velocity (Peak Vel), ratio of E-wave to E′-wave (E/E′), and Tei index = (IVCT + IVRT)/MVET were assessed by serial echocardiography in mice in each group; data are expressed as mean ± SD from 10 animals. **p < .01 vs. control group; #*p <* .05, ##*p <* .01 vs. DOX group; &*p <* .05 vs. Rg3 group. (G–I) The levels of lactate dehydrogenase (LDH), CK, and CK isoenzyme-MB (CK-MB) in mouse serum in each group, determined using an automatic biochemical analyzer (*n* = 10 per group). **p < .01 vs. control group, ##*p <* .01 vs. DOX group; &*p <* .05, &&*p <* .01 vs. Rg3 group. (J, K) ELISA results for ANP and BNP (*n* = 10 per group), **p < .01 vs. control group; #*p <* .05, ##*p <* .01 vs. DOX group; &*p <* .05 vs. Rg3 group. (L–P), Expression of systolic function-related genes (β-MHC, α-SKA, ANP, BNP, ANF) in adult cardiac myocytes from mice in each group. There was significant dysregulation of genes including classic markers of heart failure in the DOX group. This was improved by P-Rg3, resulting in ∼9-fold downregulation of β-MHC, ∼6-fold downregulation of α-SKA, ∼2-fold downregulation of ANP, ∼3-fold downregulation of BNP, and a ∼3-fold decrease in the expression of cardiac ANF. Data are presented as mean ± SD from three independent experiments. **p < .01 vs. control group; #*p <* .05, ##*p <* .01 vs. DOX group; &*p <* .05, &&*p <* .01 vs. Rg3 group.

We examined the effect of the treatment with P-Rg3 using a mouse model of DIC by M-mode color Doppler echocardiography (Figure 2(A) and Supplementary Figure 1). The ejection fraction (EF %) and fractional shortening (FS %) were significantly increased in the mice given P-Rg3 treatment compared with those in the mice given DOX alone (Figure 2(B,C)). On left ventricle outflow tract echocardiography, the peak aortic blood velocity (Vel) was significantly decreased in the DOX-treated group, but not in the P-Rg3 group (Figure 2(D)). In the P-Rg3 group, thee evaluation of the ratio of E-wave to E′-wave (E/E′) as an index of left ventricular diastolic function showed an apparent increase compared with that in the DOX group (Figure 2(E)). The Tei index in the DOX group, reflecting clinical heart function (Bruch et al., 2000), increased compared with that in the control group, which was attenuated by treatment with P-Rg3 (Figure 2(F)). We determined the effects of different interventions on the cardiac membrane integrity by estimating biochemical markers, such as LDH, CK, and CK-MB (Thollon et al., 1995). As expected, the levels of these markers were significantly increased in the DOX mice, indicating severe damage to the myocardium. The P-Rg3 group exhibited significant reductions in the myocardial levels of LDH, CK, and CK-MB compared with those in the DOX group, indicating the preservation of cardiac membrane integrity (Figure 2(G–I)). To characterize the molecular changes associated with the heart failure phenotype, we measured the levels of ANP and BNP in blood serum. Compared with the DOX group, the P-Rg3 group showed significantly decreased ANP and BNP levels (Figure 2(J,K)). The DOX heart underwent ventricular remodeling, characterized by increasing expression of heart cardiac failure (fetal) marker genes including ANP, ANF, BNP, α-SKA, and β-MHC. To determine the effect of P-Rg3 on gene expression of established molecular markers in pathological cardiac damage, we measured these in all groups. Cardiac expression of fetal genes returned to near-baseline values of control mice when mice were administered P-Rg3, but not in DOX group (Figure 2(L–P)). As demonstrated by these data, Rg3 or P-Rg3 attenuated DIC, more importantly; P-Rg3 had a more prominent protective effect than Rg3.

### P-Rg3 was required for the maintenance of normal cardiac structure and function during DIC

**Figure 3.**
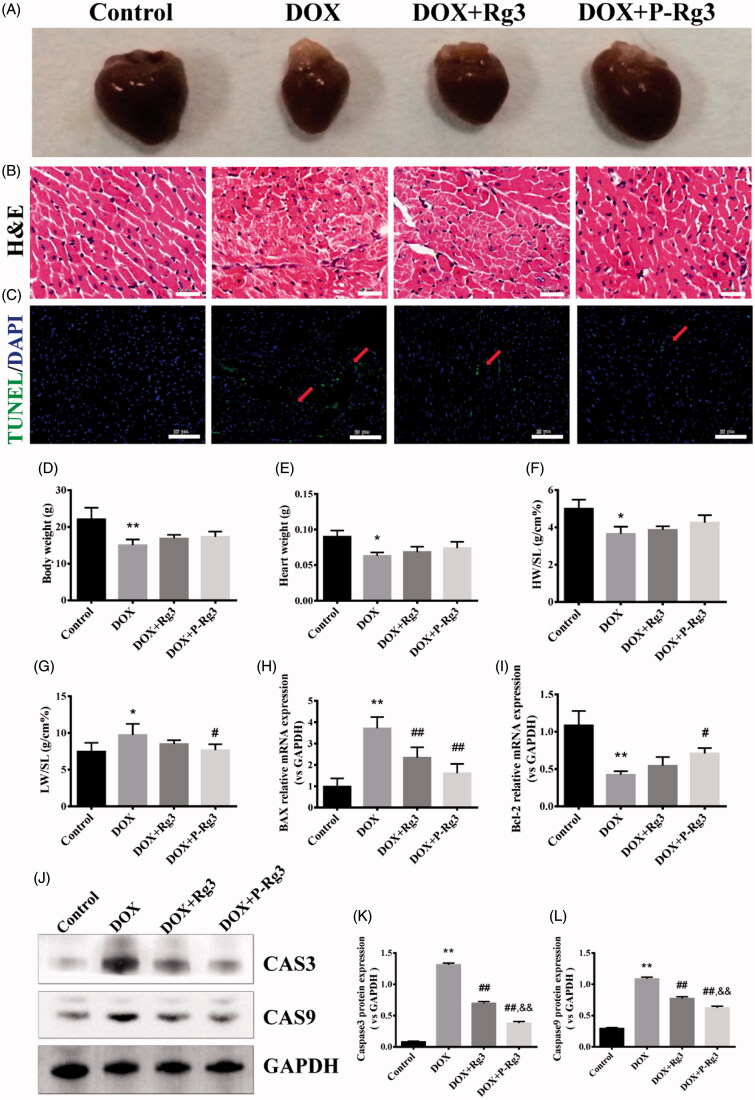
P-Rg3 prevented adverse remodeling and cardiac myocyte apoptosis induced by DOX. (A) Representative images showing gross cardiac morphology of mice in each group. (B) H&E staining of transverse sections. Scale bars, 50 μm. (C) TUNEL staining was performed on myocardial sections from mouse heart of each group. Apoptotic nuclei were visualized by green fluorescence. Nuclei were identified as blue [DAPI (4′, 6-diamidino-2-phenylindole)]. Scale bar, 25 μm. (D–G) Bar graphs showing quantitative data for body weight, heart weight, heart weight/shankbone length ratios (HW/SL), and lung weight/shankbone length ratios (LW/SL), *n* = 10. (H) and (I) Expression of apoptosis-related genes [Bcl-2-associated X protein (Bax) and Bcl-2] in adult cardiac myocytes from mice in each group. Data are presented as mean ± SD from three independent experiments. (J) Representative Western immunoblots for caspase 3 and caspase 9. (K) and (L), Bar graph showing corresponding quantitative data of caspase 3 and caspase 9. Data are presented as mean ± SD from three independent experiments. *p < .05, **p < .01 compared with the control group; #*p <* .05, ##*p <* .01 compared with the DOX group; &*p <* .05, &&*p <* .01 compared with the Rg3 group.

To determine the structural basis for the abnormal cardiac function caused by DOX and the therapeutic effects of P-Rg3, we performed gross and histological analyses of mouse hearts after DIC and P-Rg3 therapeutic effects. Gross pathological changes were also apparent in the hearts of mice in each group. P-Rg3 obviously inhibited the decrease of heart volume caused by DOX (Figure 3(A)). DOX mice exhibited decreases in heart weight, body weight, and the ratio of heart weight to shankbone length, indicating atrophy of the heart. In response to P-Rg3 treatment, these indices tend to increase, but this did not reach statistical significance compared with the levels in the DOX group (Figure 3(A,D–F)). DOX mice also had elevated lung weight/shankbone length ratio compared with the control group, indicative of pulmonary edema caused by cardiac defects (Bocchi et al., 1994), and after P-Rg3 treatment, this index significantly increased (Figure 3(G)). Staining pictures revealed that the DOX group had marked edema and cavitation; this damage was reduced after P-Rg3 treatment (Figure 3(B) and Supplementary Figure 2) Next, we measured apoptosis using the TUNEL assay. In the DOX group, apoptosis occurred; however, P-Rg3 was able to decrease the level of apoptosis compared with that in the DOX group (Figure 3(C)). To verify the anti-apoptotic effect of P-Rg3, we detected the expression change at the gene and protein levels for each group. P-Rg3 decreased the mRNA expression of Bax and increased that of Bcl-2 compared with the levels in the DOX group (Figure 3(H,L)); P-Rg3 decreased caspase 3 and caspase 9 protein expression compared with that in the DOX group (Figure 3(J–L)). Taken together, these findings suggest that P-Rg3 is essential for the maintenance of normal cardiac structure and anti-apoptosis during DIC, and its efficiency is higher than that of Rg3.

### P-Rg3 prevented mitochondrial function loss in DOX hearts

**Figure 4.**
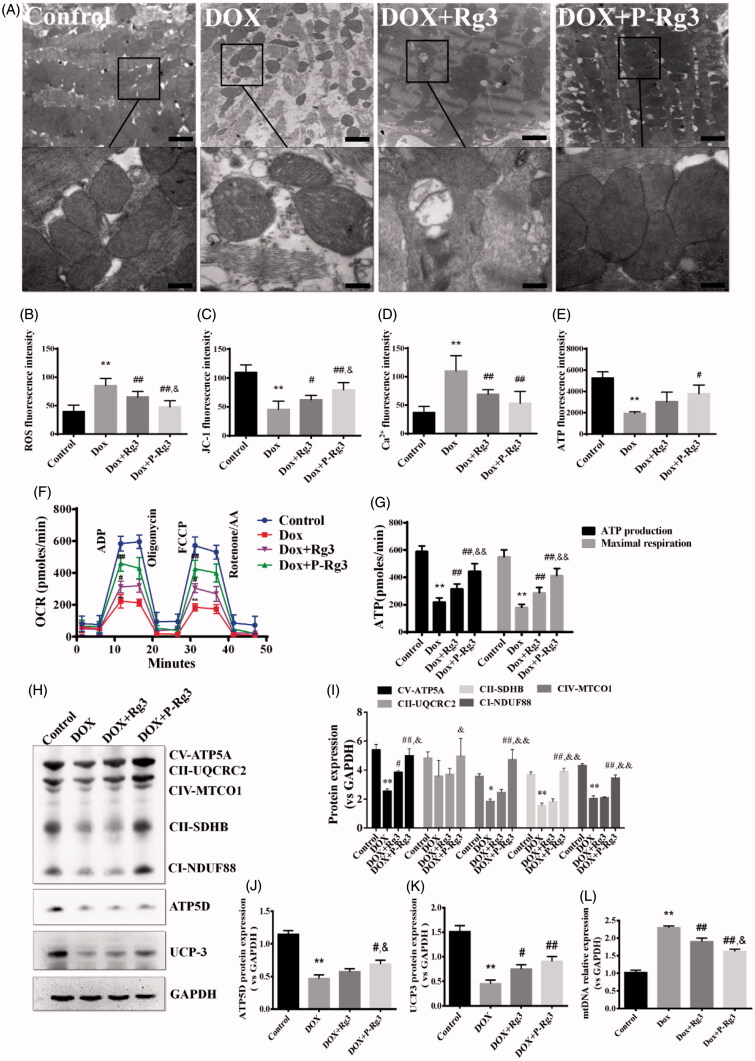
Assessment of mitochondrial function in mice of each group. (A) Ultrastructural analysis of mitochondrial integrity by transmission electron microscopy. Scale bar, top: 500 nm, bottom: 100 nm. (B) The ROS in myocardial mitochondria using DCFH-DA fluorescent probe detection kit. (C) Mitochondrial membrane potential (ΔΨm) measurement with the JC-1 probe. (D) Mitochondrial calcium overload (Ca2+) measurement with the vital dye calcein 4-AM. (E) Adenosine triphosphate (ATP) content, an indicator of mitochondrial function, was measured with ATP probe. *n* = 6, Data are presented as mean ± SD, **p < .01 compared with the control group; #*p <* .05, ##*p <* 0.01 compared with the DOX group; &*p <* .05, compared with the Rg3 group. (F, G) Oxygen consumption rate(OCR) in mitochondria isolated from the hearts of various groups was measured with a Seahorse metabolic analyzer, following the addition of the ADP, ATP coupler oligomycin, then the electron transport chain(ETC) accelerator FCCP, and then rotenone/antimycin A. ATP production levels and mitochondrial maximal respiration were then quantified. *n* = 3. Data are presented as mean ± SD, **p < .01 compared with the control group; ##*p <* .01 compared with the DOX group; &&*p <* .01 compared with the Rg3 group. (H) Representative Western immunoblots for oxidative phosphorylation (OXPHOS) complexes, ATP synthase delta 5 (ATP5D), and mitochondrial uncoupling protein 3 (UCP3). (I–K) Bar graph showing corresponding quantitative data for Western blotting. Data are presented as mean ± SD from three independent experiments, *p < .05, **p < .01 compared with the control group; #*p <* .05, ##*p <* .01 compared with the DOX group; &*p <* .05, &&*p <* .01 compared with the Rg3 group. (L) Total DNA from heart tissues of mice from each group was purified and subjected to quantitative PCR with specific primers for murine mitochondrial DNA (mtDNA). Data are presented as mean ± SD from three independent experiments, **p < .01 compared with the control group; ##*p <* .01 compared with the DOX group; &*p <* .05 compared with the Rg3 group.

To test our hypothesis that P-Rg3 exerts its cardioprotective function by preserving mitochondrial integrity and function, we measured mitochondrial structure, mitochondrial DNA copy number, and mitochondrial function in hearts. In normal myocardium, mitochondria were aligned in well-preserved rows between the longitudinally oriented cardiac myofibrils. The abundance of mitochondria in the DOX group appeared significantly altered, and DOX destroyed mitochondrial arrays and aggregated swollen mitochondria with cristae. In contrast, P-Rg3 alleviated mitochondrial swelling and crista reduction, and maintained mitochondrial arrays. In the P-Rg3 group, the state of mitochondria was close to that in the normal group. The protective effect of Rg3 was inferior to that of P-Rg3 (Figure 4(A)). P-Rg3 decreased the levels of ROS and calcium overload, while it increased mitochondrial ΔΨm, compared with those in the DOX group (Figures 2(B) and 4(C,D)). We also measured ATP content, an important indicator of mitochondrial function. In DOX hearts, ATP content significantly decreased, while it increased after P-Rg3 treatment (Figure 4(E)). We further measured OCR in cardiomyocytes isolated from mouse heart. DOX decreased the ATP coupler response and electron transport chain accelerator response. DOX-treated mice exhibited mitochondrial respiratory chain defects including reduced ATP production levels and mitochondrial maximal respiration (MMR) capacity indicating that mitochondrial respiration was severely impaired. Furthermore, P-Rg3 restored them, with its effect in this regard being superior to that of Rg3 (Figure 4(F,G)). We also examined the protein expression level of the OXPHOS complexes by Western blotting. We observed an overall reduction in the subunits of respiratory complexes in the DOX group; its content of subunits increased after the administration of P-Rg3 (Figure 4(H,I)). We observed decreases in the mitochondrial uncoupling protein (UCP3) and ATP synthase delta 5(ATP5D) levels in the DOX group, which were increased in the P-Rg3 group (Figure 4(H,J–K)). Quantitative PCR showed that mitochondrial DNA copy number (mtDNA) increased in DOX hearts, while P-Rg3 decreased mtDNA (Figure 4(L)). These findings indicate that P-Rg3 is more effective than Rg3 at protecting heart against DOX cardiotoxicity, including the loss of mitochondrial integrity, mitochondrial swelling, ultrastructural derangement, and reduced ATP production.

### P-Rg3 increased myocyte viability and decreased apoptosis *in vitro* after DOX treatment

**Figure 5.**
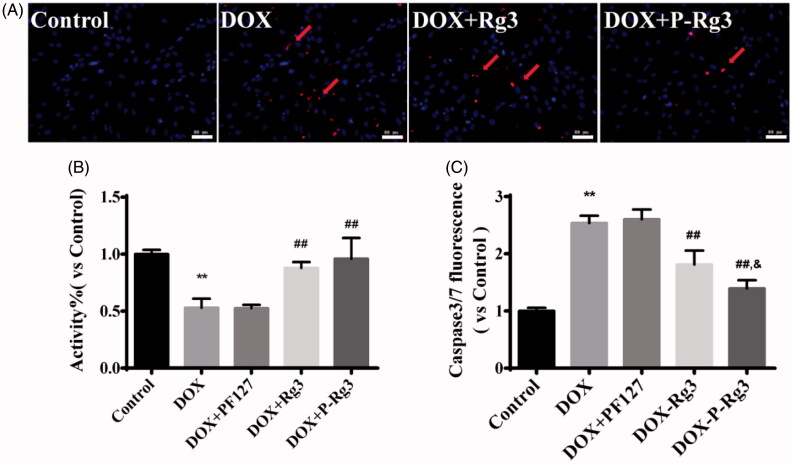
P-Rg3 prevented adverse cardiac myocyte apoptosis induced by DOX *in vitro*. (A) Representative images are of TUNEL staining of H9C2 cells from various groups; apoptotic nuclei are visualized as red fluorescence. Nuclei are identified as blue [DAPI (4′, 6-diamidino-2-phenylindole)]. Scale bar, 25 μm (*n* = 3 per group). (B) Cell viability of H9C2 cells was measured by MTT, *n* = 6. (C) Cell apoptosis was measured by caspase 3/7 probe, *n* = 6. Data are expressed as mean ± SD, **p < .01 compared with the control group; ##*p <* .01 compared with the DOX group; &*p <* .05, compared with the Rg3 group.

The TUNEL assay showed that P-Rg3 could decrease the level of apoptosis compared with that in the DOX group (Figure 5(A)). To determine whether cell death in H9C2 cells was driven by apoptosis, caspase 3/7 activities were assessed for all groups. The fold increase in caspase 3/7 activity of P-Rg3 was much lower than that of the DOX and Rg3 groups (Figure 5(C)). The MTT assay results showed that P-Rg3 protects H9C2 cells against the DOX-induced decrease in cell activity (Figure 5(B)).

### P-Rg3 inhibited mitochondrial dysfunction caused by DOX in H9C2 cells

**Figure 6.**
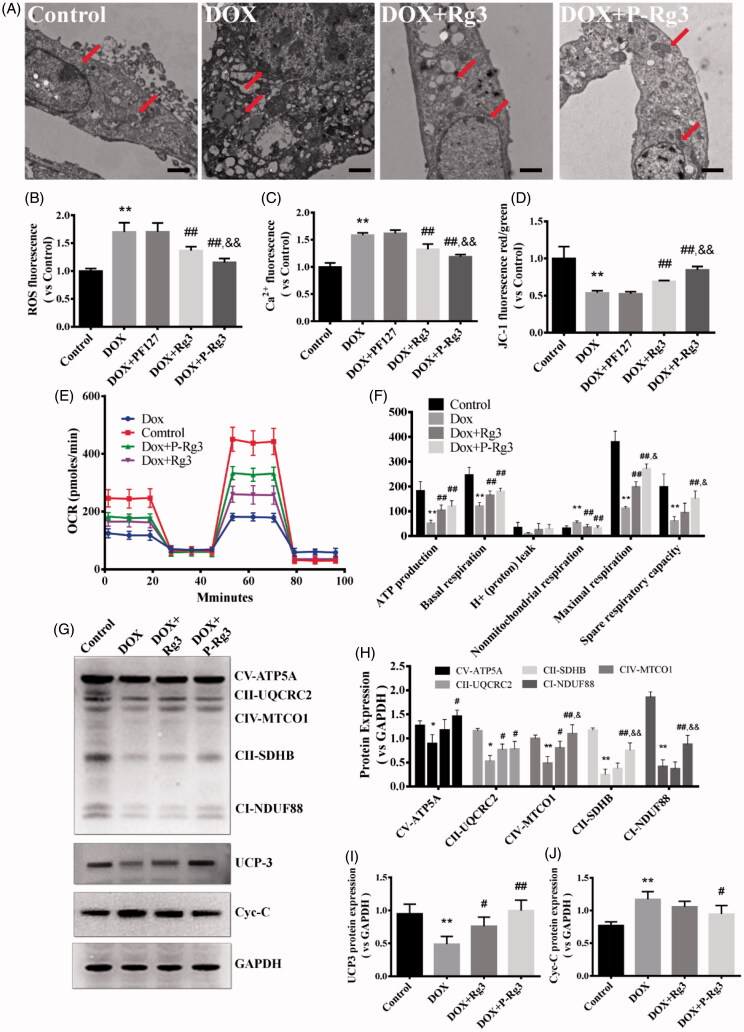
P-Rg3 inhibited mitochondrial dysfunction caused by DOX in H9C2 cells. (A) Ultrastructural analysis of H9C2 cells by transmission electron microscopy. Scale bar, 500 nm. (B) The ROS in H9C2 cells were tested using DCFH-DA fluorescent probe detection kit. (C) Mitochondrial calcium overload (Ca2+) was measured with the vital dye calcein-AM. D, Mitochondrial membrane potential (ΔΨm) was measured with the JC-1 probe. *n* = 6. Data are expressed as mean ± SD. **p < .01 compared with the control group; ##*p <* .01 compared with the DOX group; &&*p <* .01 compared with the Rg3 group. (E, F) OCR in H9C2 cells of various groups was measured with a Seahorse metabolic analyzer (details are provided in F). *n* = 3 for each group. (G) Representative Western immunoblots for OXPHOS complexes, Cyc-C, and UCP3. (H–J) Bar graph showing corresponding quantitative data. Data are presented as mean ± SD from three independent experiments, *p < .05, **p < .01 compared with the control group; #*p <* .05, ##*p <* .01 compared with the DOX group; &*p <* .05, &&*p <* .01 compared with the Rg3 group.

**Figure 7.**
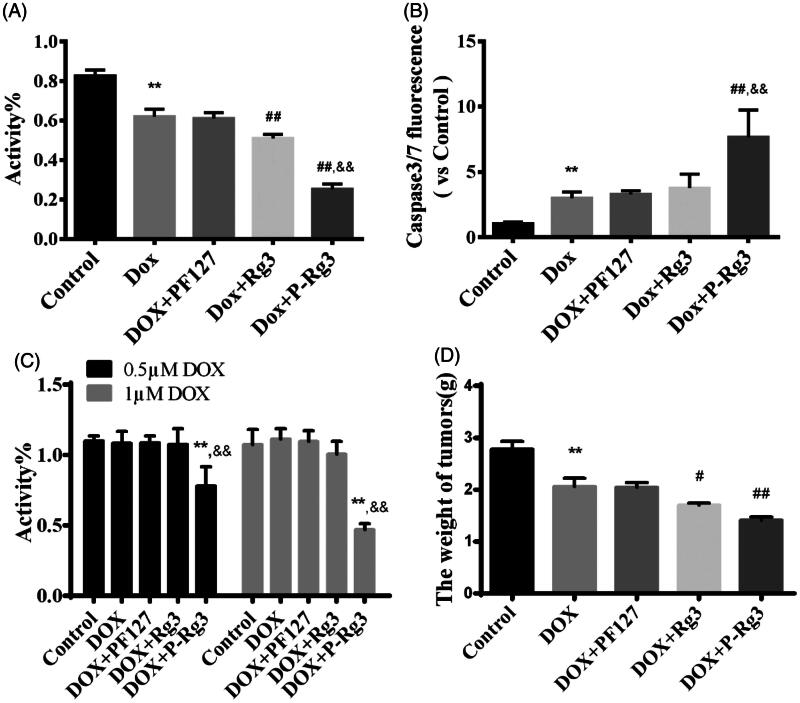
P-Rg3 increased the inhibitory effect of DOX in breast cancer. (A) Viability of 4T1β cells was measured by MTT, *n* = 6. (B) Cell apoptosis was measured by caspase 3/7 probe in 4T1β cells, *n* = 6. (C) Viability of MDA-MB-231 cells was measured by MTT, *n* = 6. (D) After treatment for 14 days, the tumor weight was examined in each group, *n* = 6. Data are expressed as mean ± SD, **p < .01 compared with the control group; #*p <* .05, ##*p <* .01 compared with the DOX group; &&*p <* .01 compared with the Rg3 group.

TEM images showed that DOX-induced cell hypertrophy and subcellular organelle damage, particularly in nuclear expansion, and mitochondrial, and endoplasmic reticulum edema. After P-Rg3 treatment, mitochondria, and endoplasmic reticulum exhibited an intact morphology, which resembled the findings in the control group (Figure 6(A)). DOX also increased mitochondrial ROS generation, while P-Rg3 prevented this from happening (Figure 7(B)). P-Rg3 resulted in a decrease of calcium overload compared with that in the DOX group (Figure 6(C)). DOX also resulted in significant mitochondrial membrane depolarization in H9C2 cells, and P-Rg3 prevented this from happening (Figure 6(D)). Compared with DOX cells, the P-Rg3-treated cells exhibited marked increases in basal respiration, ATP production, MMR capacity, and mitochondrial spare respiratory capacity (Figure 6(E,F)). The amounts of proteins involved in oxidative phosphorylation, as assessed by immunoblotting, showed a trend similar to that *in vivo* (Figure 6(G–J)).

### P-Rg3 sensitized DOX-mediated killing of breast cancer cells by increasing apoptotic activities

The MTT assays showed that P-Rg3 increased the anticancer potency of DOX in 4T1β and MDA-MB-231 cells, indicating that P-Rg3 has chemosensitizing effects when used in combination with DOX (Figure 7(A,C) and Supplementary Figure 3). Blank Pluronic micelles had no adverse effects on cell viability. To determine whether cell death in 4T1β cells is driven by apoptosis, caspase 3/7 activity was assessed for all treatments. P-Rg3 increased the caspase 3/7 activity in 4T1β cells, which was much higher than those in the DOX and Rg3 groups (Figure 7(B)). Tumor weight in the P-Rg3 group was much lower than that in the DOX group (Figure 7(D) and Supplementary Figure 4).

## Discussion

The clinical application of doxorubicin is associated with various adverse reactions, including cardiotoxicity, myelosuppression, and digestive system injury (Lipshultz et al., 2015). These adverse reactions seriously affect the quality of life after chemotherapy and severely restrict the clinical application of doxorubicin (Giordano et al., 2012). Ginsenoside Rg3 is a tetracyclic triterpenoid saponin extracted from ginseng, which has a variety of pharmacological activities including cardiovascular protection; increased detoxification and immunity; and the inhibition of tumor cell proliferation, invasion, and metastasis (Smith et al., 2014; Cheng et al., 2016a; Kim et al., 2017). However, the low solubility and oral bioavailability of Rg3 limit its therapeutic effect. Similarly (Yang et al., 2012), such limitations exist in many other natural products (Hollman et al., 1997; Walle, 2011; Liu et al., 2013). The using Rg3 for inducing healing and inhibiting scar hyperplasia has been reported by researchers (Cheng et al., 2013b; Cheng et al., 2014; Sun et al., 2014; Cheng et al., 2016a), while there is limited achievement on the Rg3 mitigating doxorubicin-induced cardiotoxicity. Our previous study showed that Rg3 could alleviate doxorubicin toxicity by improving endothelial dysfunction caused by oxidative stress (Wang et al., 2015a), but the deep-seated mechanism of DIC requires further study. In recent similar studies, Cote et al. reported that combinatorial resveratrol and quercetin polymeric micelles could mitigate DIC (Cote et al., 2015); Cheng et al. reported that epigallocatechin-3-O-gallate based catechin-based polyion complex micelles could overcome DOX induced cardiotoxicity and multidrug resistance (Cheng et al., 2016b); and another study by El-Ashmawy et al. shown that combination therapy with thymoquinone nanomatrix and DOX could limit its cardiac toxicity and enhance anti-cancer activity (El-Ashmawy et al., 2017); however, these studies did not include any pharmacokinetic profile or information on the mechanism of heart protection.

Here, we encapsulated Ginsenoside Rg3 using a widely accepted triblock block copolymer Pluronic F127. Ginsenoside Rg3 was encapsulated in the hydrophobic core of Pluronic F127 with the hydrophilic end exposed outside to increase the solubility of the P-Rg3. As we had expected, the solubility of P-Rg3 was increased by 50–200 times compared with that of the Rg3 monomer, and we speculate that encapsulation can promote stomach and intestinal absorption of Rg3 (Zhu et al., 2013). Approximately 80% of Rg3 was released from P-Rg3 after 72 h in an aqueous environment. P-Rg3 showed a sustained release effect, which could significantly increase the circulation time of drugs. *In vivo* pharmacokinetic studies showed that P-Rg3 plasma concentration reached the peak value much faster than Rg3, and the AUC of P-Rg3 was approximately 3.2 times higher than that of Rg3 in rat plasma. The encapsulation of Rg3 could enhance the rate and amount of Rg3 absorption, which further improved its bioavailability. The improvement of oral bioavailability of Rg3 not only prolonged survival time but also improved survival rate compared with that for Rg3 monomers. In addition, the small size of P-Rg3 (less than 100 nm) enables them to avoid absorption into the reticuloendothelial system (RES), which also prolong the drug circulating time and promoted the drug distribution in the tumors (Yokoyama, 2005; Chu et al., 2014).

**Figure 8.**
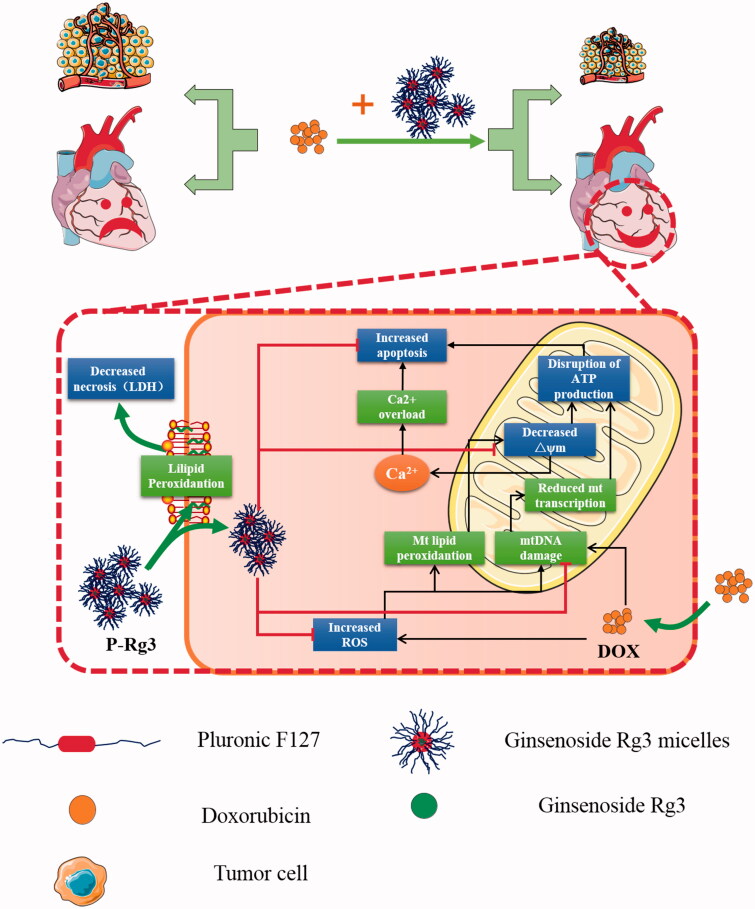
A schematic illustration showed that Ginsenoside Rg3 micelles mitigate doxorubicin-induced cardiotoxicity (DIC) and enhance its anticancer efficacy.

Mitochondria are densely distributed in heart tissue. DOX and its metabolites can directly damage the structure and function of mitochondria, which is one of the primary reasons for DIC. Therefore, the protection of mitochondria from DOX damage is an effective way to alleviate DIC. The mechanisms of DIC comprise several interrelated subsets, including: generation of ROS, subsequent mitochondrial membrane damage, and calcium overload, which lead to decreased ATP production (Octavia et al., 2012; Zhang et al., 2012; Ichikawa et al., 2014; Burridge et al., 2016). The generation of ROS by redox cycling of doxorubicin within cardiomyocytes causes mitochondrial dysregulation, lipid peroxidation, and DNA damage (Zhang et al., 2012; Khiati et al., 2014). We found that P-Rg3 could significantly inhibit the production of ROS induced by doxorubicin and its metabolites. Doxorubicin binding irreversibly to mitochondrial membrane leads to a decrease of ΔΨm (Monteiro et al., 2013). The loss of ΔΨm and increased ROS may disrupt respiratory chain activity, which would further decrease ATP generation (Monteiro et al., 2013; Buondonno et al., 2016). Here, it is worth noting that P-Rg3 simultaneously increased the production and maximum reserve capacity of ATP both *in vivo* and *in vitro*. Additionally, P-Rg3 inhibited mitochondrial DNA damage and DNA-damage-induced apoptosis. DOX and its major metabolite are potent calcium-release channel activators and can induce Ca^2+^ efflux from the sarcoplasmic reticulum, which causes sarcomeric disarray and myofibril deterioration (Peters et al., 2016; Zhao et al., 2017). Our results showed that P-Rg3 could maintain homeostasis of the sarcoplasmic reticulum by inhibiting calcium permeability, which reduced the cardiotoxicity of doxorubicin. The cardiotoxicity of doxorubicin leads to ultrastructural change including cytoplasmic vacuole formation, mitochondrial disarrangement, mitochondrial swelling, mitochondrial ridge, and membrane disassembly. Impaired mitochondrial structure leads to irreversible damage of mitochondrial respiratory function, which further causes systolic and diastolic dysfunction. We found that P-Rg3 significantly inhibited DOX-induced ultrastructural damage and improved heart function (Figure 8).

We also evaluated the antitumor ability of doxorubicin combined with P-Rg3. Notably, P-Rg3 demonstrated higher antitumor efficacy than Rg3, which may have been because of high permeability and retention effects of P-Rg3. More importantly, we used MDA-MB-231 doxorubicin-resistant breast cancer cell lines to assess the pharmacological function of P-Rg3. Only P-Rg3 could induce sensitization to doxorubicin and antagonize the drug resistance of breast cancer cells, the mechanism of which is to be studied in further research.

In conclusion, Rg3 aqueous solubility and oral bioavailability were markedly improved by Pluronic F127 encapsulation. P-Rg3 alleviated cardiotoxicity and mitochondrial damage introduced by doxorubicin. P-Rg3 co-administered with DOX was capable of improving the efficacy of DOX against cancer, and overcame the MDR in breast cancers. This study indicates that polymeric micellar encapsulated natural product co-administered with chemotherapeutic agents was more effective than the drug alone, increasing efficacy, and decreasing toxicity.

## Supplementary Material

IDRD_Guanwei__et_al_Supplement_Content.docx
