# Supplementary material for: Ginsenoside Rg3 micelles mitigate doxorubicin-induced cardiotoxicity and enhance its anticancer efficacy
Source: Drug Deliv. 2017 Oct 24;24(1):1617–30. doi: 10.1080/10717544.2017.1391893 (PMC8241051; doi:10.1080/10717544.2017.1391893)
Supplement: IDRD_Guanwei__et_al_Supplement_Content.docx [file IDRD_A_1391893_SM1890.docx]

**SUPPLEMENTARY INFORMATION**

**Ginsenoside** **Rg3 micelles mitigate doxorubicin-induced** **cardiotoxicity** **and enhance its anticancer efficacy**

Lan Li ^1,2^，Jingyu Ni ^1,2^，Min Li ^1,2^，Jingrui Chen ^1,2^，Lifeng Han ^2^，Yan Zhu ^2^，Deling Kong^3^, Jingyuan Mao^1^，Yi Wang^4^，Boli Zhang^2^，Meifeng Zhu*^3^，Xiumei Gao*^2^，Guanwei Fan*^1,2^

1. First teaching hospital of Tianjin University of Traditional Chinese Medicine, Tianjin 300193, China.

2. State Key Laboratory of Modern Chinese Medicine, Tianjin University of Traditional Chinese Medicine, Tianjin 300193, China.

3. State Key Laboratory of Medicinal Chemical Biology, Key Laboratory of Bioactive Materials of Ministry of Education, College of Life Science, Nankai University, Tianjin 300071, China.

4. College of Pharmaceutical Sciences, Zhejiang University, Hangzhou, Zhejiang China.

*Correspondence:

Tel.:﹢862259596163; Fax:﹢862227412653

E-mail:

Prof. Guanwei Fan, [fgw1005@hotmail.com](mailto:fgw1005@hotmail.com),

Prof. Xiumei Gao, [gaoxiumei@tjutcm.edu.cn](mailto:gaoxiumei@tjutcm.edu.cn),

Dr. Meifeng Zhu, [zhumeifeng2013@163.com](mailto:zhumeifeng2013@163.com)

**Supplementary methods**

**Pharmacokinetic study**

**LC-MS conditions:** The LC/MS analysis was performed on an Ultimate 3000 UHPLC coupled with Q-Exactive mass spectrometer by HESI source (Thermo Fisher Scientific, San Jose, CA, USA). Chromatographic separation was achieved on an Agilent Eclipse plus C_18_ RRHD column (2.1 × 50 mm, 1.8 μm). The mobile phase was water (containing 0.05% acetic acid, *v*/*v*) (A) and ACN (D). Gradient elution was set as follows: 20-100% D from 0 to 4.0 min, 100-20% D from 4.0 to 4.5 min, and 20% D from 4.5 to 5.0 min. The flow rate was set at 0.4 mL/min and the injection volume was 2 μL. The column temperature was maintained at 40 ºC. Source parameters were optimized as follows: SIM scanning in negative mode was used; the source spray voltage was set at 2.8 kV; capillary and Aux gas heater temperature were 320 and 350 ºC, respectively; S-lens RF level, sheath gas (N_2_), and auxiliary gas (N_2_) were 50, 35 and 10 arbitrary units, respectively. Quasi-molecule ions of [M−H] ^–^ were selected at *m/z* 783.49002 for ginsenoside Rg3 and *m/z* 799.48493 for the IS.

**Stock solutions:** The primary stock solutions of ginsenoside Rg3 and ginsenoside Rg1 was prepared by dissolving them in methanol at the concentrations of 1mg/mL and were stored at 4 ºC in refrigerator until analysis. Calibration curve and QC working solutions were prepared by appropriate dilution of the primary stock solutions in methanol. Working solution of IS was dissolved in methanol at a concentration of 500 ng/mL. QC samples were prepared with the three concentrations of ginsenoside Rg3 at high (100 ng/mL), medium (10 ng/mL), and low level (1 ng/mL).

**Sample preparation:** For construction of the calibration curve, 10 μL of diluted standard working solutions and IS were spiked into 100 μL rat plasma, and then 400 μL of ACN was added into the mixture. The mixture was vortexed for 5 min and centrifuged at 14,000 × g for 10 min. 480 μL of the supernatant was transferred into another 1.5 mL centrifuge tube and evaporated to dryness by nitrogen gas. The residue was dissolved in by 100 μl methanol and centrifuged at 14,000 × g for 10 min. 2 μL aliquot of the supernatant was analyzed using LC-MS system.

**Linearity:** The calibration curve was assessed by plotting the peak response ratios of ginsenoside Rg3/IS against their correspond concentration. The final concentration levels of calibration standards were 0.5, 1.0, 5.0, 10.0, 100.0, 500.0 ng/mL. The correlation coefficient of the calibration curves generated during each analyte was more than 0.99. The lowest limit of reliable quantification for each analyte was regarded as the concentration of the LLOQ.

**Precision and accuracy:** The precision and accuracy were calculated at the three QC samples. The QC samples were run in different days. The accuracy as RE was to be within 85-115%. The intra-day and inter-day precision should be within ± 15% with expressed as RSD.

**Extraction recovery and matrix effect:** The recoveries was evaluated at six replicates of each QC samples (1.0, 10.0 and 100.0 ng/mL) in rat plasma. The recoveries were determined by comparing the peak response of before extraction spiked QC samples with corresponding after extraction spiked QC samples concentration levels.The matrix effect on the analytes were evaluated by comparing the peak response ratio of post-extraction blank plasma at concentrations of QC samples with the areas obtained by the corresponding standard solutions. Three different concentrations were evaluated by analyzing the calibration curve.

**Measurements of mitochondrial membrane potential (ΔΨm)**

Mitochondrial inner membrane potential (ΔΨm) was monitored by applying the fluorescent dye JC-1 (Beyotime, China). JC-1 indicated mitochondrial polarization by shifting its fluorescence emission from green (525 nm) to red (590 nm). Using the dual fluorescence characteristic of this dye, the changes in mitochondrial ΔΨm can be assessed by comparing the ratios of red/green fluorescence emission. Opening of mitochondria permeability pore (mPTP) can cause the loss of ΔΨm, which results in a decrease in the red/green optical density ratio.

**Real-time reverse transcriptase polymerase chain reaction (RT-PCR) quantification and** **mitochondrial DNA quantification**

PT-PCR analysis was performed for gene expression and mitochondrial DNA quantification on tissue extract, as reported previously. Quantification of mitochondrial DNA was performed by quantitative PCR using the following primers: F, 5′-GCCCCAGATATAGCATTCCC-3′; and R, 5′-GTTCATCCTGTTCCTGCTCC-3′. The fold induction for each well was calculated by using the 2− (ΔΔCT) formula.

| Gene |  | Sequence |
| --- | --- | --- |
| ANP | primer F | GAAAAGCAAACTGAGGGCTCTG |
|  | primer R | CCTACCCCCGAAGCAGCT |
| BNP | primer F | CTGCTGGAGCTGATAAGAGA |
|  | primer R | TGCCCAAAGCAGCTTGAGAT |
| ANF | primer F | GGAGGAGAAGATGCCGGTAGA |
|  | primer R | GCTTCCTCAGTCTGCTCACTCA |
| α-SKA | primer F | CCACAGGGCTTTGTTTGA AAA |
|  | primer R | CTGAGGTACAGCTGGATGTT |
| β-MHC | primer F | GTGCCAAGGGCCTGAATGAG |
|  | primer R | GCA AAGGCTCCAGGTCTGA |
| Bax | primer F | CGGCGAATTGGAGATGAACTG |
|  | primer R | GCAAAGTAGAAGAGGGCAACC |
| Bcl-2 | primer F | TAGAGAGATGCGAGGAACCGATG |
|  | primer R | TAGAGAGATGCGAGGAACCGATG |
| GAPDH | primer F | AGGTCGGTGTGAACGGATTTG |
|  | primer R | AGGTCGGTGTGAACGGATTTG |

Abbreviations: RT-PCR, reverse-transcription polymerase chain reaction; ANP, atrial natriuretic peptide; BNP, brain natriuretic peptide; ANF, atrial natriuretic factor; α-SKA, α-smooth muscle actin; β-MHC, β-myosin heavy chain; GAPDH, glyceraldehyde-3-phosphate dehydrogenase

**Western blot analysis**

Western blot analysis was performed for protein expression on heart tissue or cell lysate extract, as reported previously. Protein extracts were resolved on denaturing SDS/PAGE gels transferred to PVDF membranes.

| **Antibody** | **Source** | **Cat#** |
| --- | --- | --- |
| Mitochondrial complex | Abcam | Ab6213 |
| UCP3 | Proteintech | [10750-1-AP](http://www.ptgcn.com/products/UCP3-Antibody-10750-1-AP.htm) |
| Cyc-C | Abcam | Ab12772 |
| ATP5D | Abcam | Ab87242 |
| Caspase3 | Proteintech | [19677-1-AP](http://www.ptgcn.com/products/CASP3-Antibody-19677-1-AP.htm) |
| Caspase9 | Proteintech | [10380-1-AP](http://www.ptgcn.com/products/CASP9-Antibody-10380-1-AP.htm) |

**Supplementary Figure 1.** **A-F,** Echocardiographic characterization of cardiac systolic function subjected to DOX induced cardiotoxicity. Data are presented as mean ± SD from three independent experiments. ^**^*p <* 0.01 compared with the control group; ^#^*p <* 0.05, ^##^*p <* 0.01 compared with the DOX group; ^&^*p <* 0.05, ^&&^*p <* 0.01 compared with the Rg3 group.

**
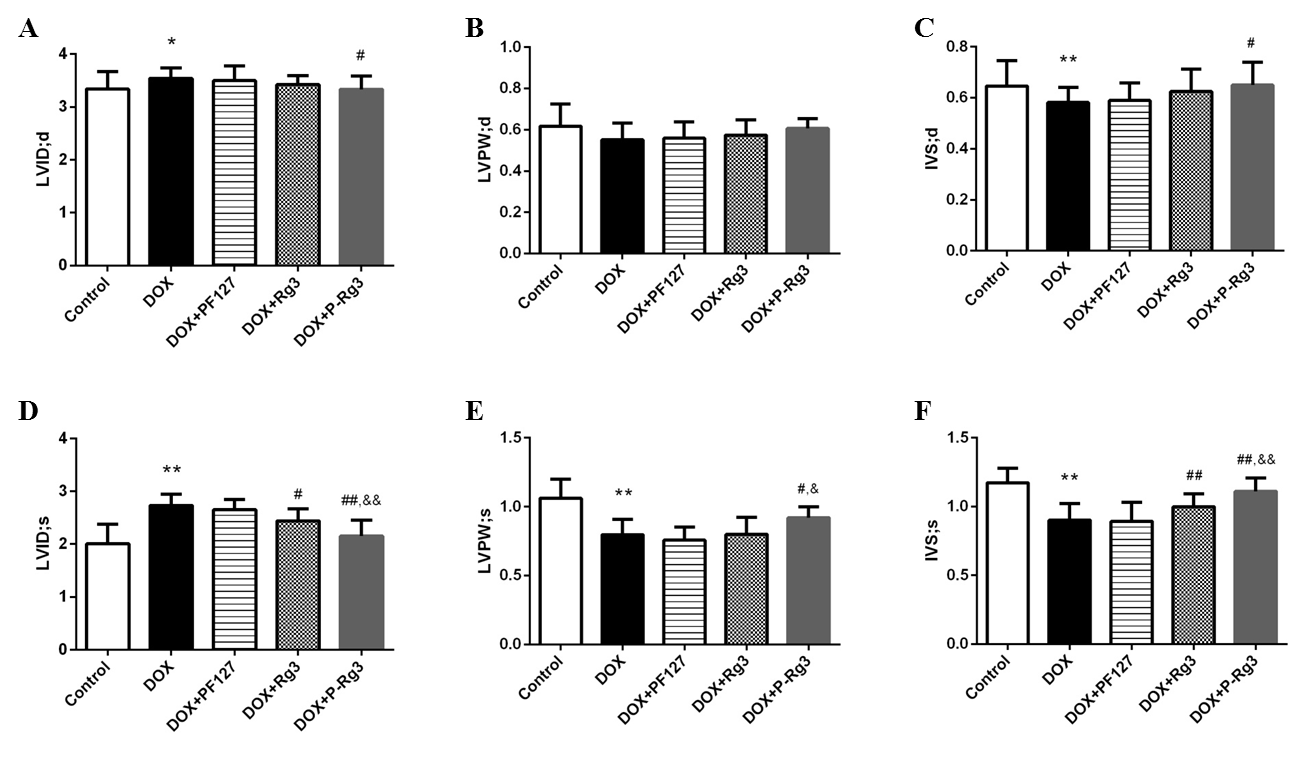
**

**Supplementary Figure 2.** Microscopic cross sections stained with wheat germ agglutinin (WGA) revealed DOX-induced increases in cell size in hearts, while the cardiomyocyte cross-sectional area was decreased in P-Rg3 hearts. Scale bars, 50 μm.

**
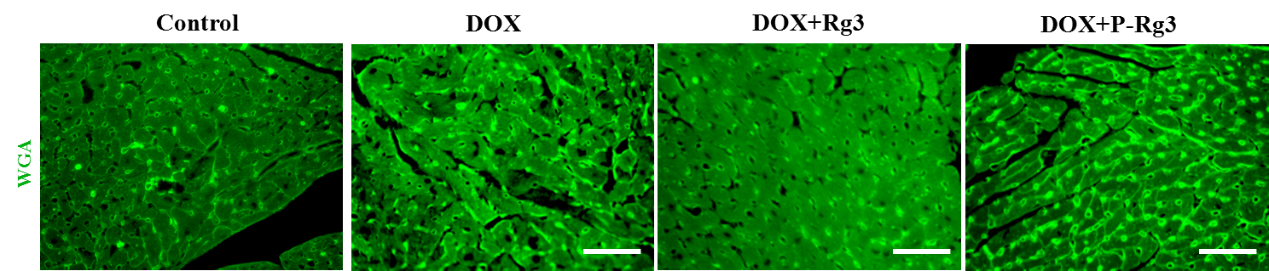
**

**Supplementary Figure 3.** WGA staining revealed P-Rg3 increased the anticancer potency of DOX in 4T1β tumos cells. Scale bars, 25 μm. **
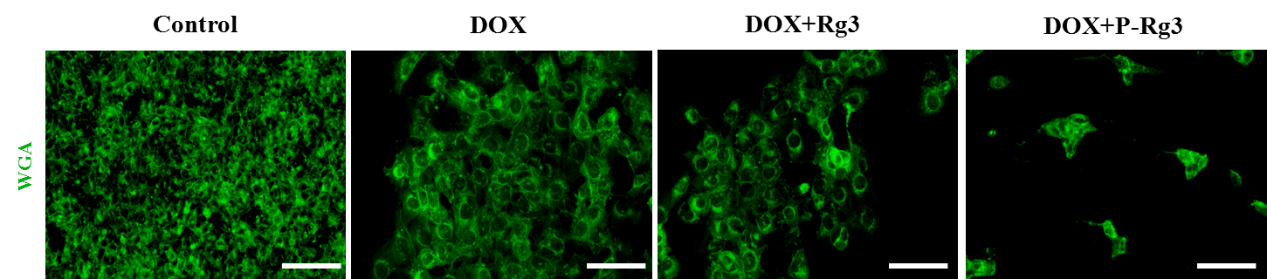
**

**Supplementary Figure 4.** The tumos volume, tumos volume/body weight and tumos weight/body weight was examined in each group, n = 6. Data are expressed as mean ± SD, ^#^*p <* 0.05 compared with the DOX group.

**
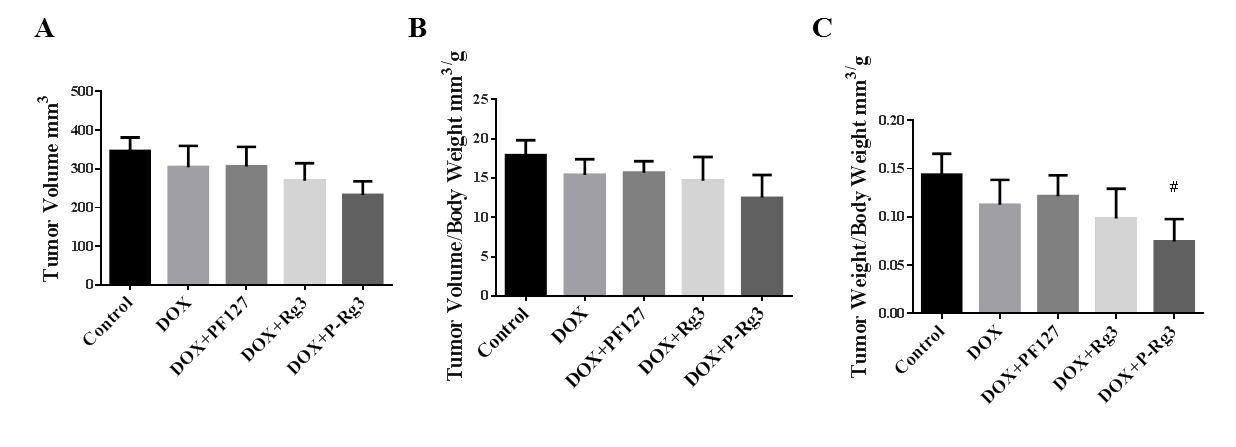
**

**Supplementary Figure 5.** Cell viability of 4T1β cells was measured by MTT, n = 6. ^**^*p <* 0.01 compared with the control group.

**
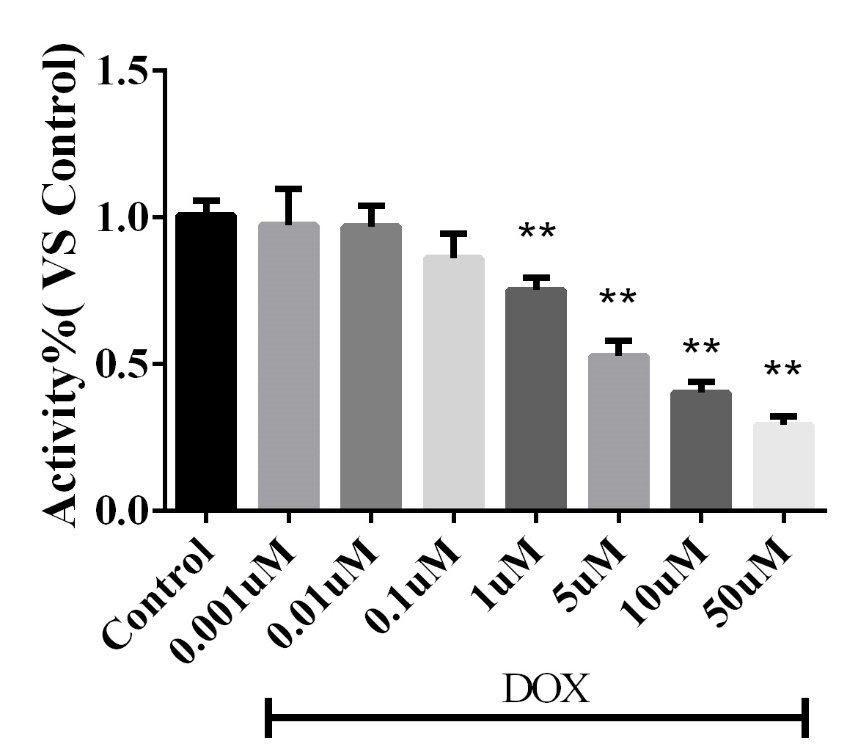
**
